# Editorial: The dark and the light side of gaming

**DOI:** 10.3389/fpsyg.2023.1349479

**Published:** 2024-01-09

**Authors:** Felix Reer, Marko Siitonen, Teresa De La Hera

**Affiliations:** ^1^Department of Communication, University of Münster, Münster, Germany; ^2^Department of Language and Communication Studies, University of Jyväskylä, Jyväskylä, Finland; ^3^Department of Media and Communication, Erasmus University Rotterdam, Rotterdam, Netherlands

**Keywords:** digital games, media effects, media use, gaming communities, video game culture

Debates about the effects of digital gaming date back to the 1980s and 1990s when first affordable home computer systems were introduced. Early research often concentrated on exploring the potential *dark side of gaming*, trying to shed light into the social discourses that established relationships between gaming and negative effects in players and gaming communities. These studies have explored, for example, the purported influence of playing violent games on aggressiveness (e.g., Cooper and Mackie, 1986) or focused on the addictive potentials of games (e.g., Fisher, 1994). However, it should be noted that some studies in these research areas have been criticized for methodological reasons and that researchers' opinions on potential negative effects of gaming differ (Drummond et al., 2018; Nielsen and Kardefelt-Winther, 2018; Pontes, 2018; Scharrer et al., 2018). In spite of these ongoing discussions, there has been a notable shift in the last decades and interest in *the light side of gaming* has increased. For example, studies have examined the positive potentials of games in terms of skill development (e.g., Bediou et al., 2023), learning (e.g., Clark et al., 2016), and stress reduction (e.g., Pallavicini et al., 2021).

With this Research Topic, we wanted to demonstrate how multifaceted and complex games and play are. Our aim was to bring together research on both positive and negative aspects related to games and play, as well as research that sheds light on the many gray areas between these two extremes.

## The dark side

Three articles of the current Research Topic focus on exploring the relationship between gaming and potential negative effects, trying to provide understanding to the dark side of games and play.

The study by Olejarnik and Romano examines how personality aspects (narcissism and self-esteem) as well as violent video game choice (classified according to the Pan European Game Information rating; PEGI) are related to different indicators of aggressiveness (anger, hostility, physical aggression, verbal aggression). Conducting a cross-sectional survey among 166 game users, the study found that violent video game choice and narcissism predict verbal aggression, while hostility is predicted by violent video game choice and lower self-esteem. The authors stress the importance of adequate age rating systems and safeguard procedures to protect younger users.

Rather than focusing on violent content of particular games, Cook et al. take a closer look on aggressive behaviors between online gamers. Their interview study takes an innovative approach by contrasting and comparing toxic behaviors on gaming platforms and on social media. The authors found that online gaming is in general perceived as more toxic than social media use. Further, trolling in gaming contexts is mainly understood as unfair behavior toward fellow players (e.g., disadvantaging one's team), while trolling on social media is defined in a broader way.

A topic that has received growing attention is the question of whether playing online games can serve as a so-called breeding ground for extremism. To prevent such developments, it is important to increase the understanding of the underlying mechanism of the radicalization of gamers. Against this background, Kowert et al. conducted three quantitative surveys and identified several relevant factors, including fusing with gamer identity (i.e., a deep alignment with gaming culture and the group of gamers), problematic personality traits (narcissism, psychopathy), individual differences (i.e., loneliness, insecure attachment), and enthusiasm for violent games (i.e., Call of Duty).

## The light side

The Research Topic also includes five articles that examine the possible positive effects of gaming.

Rüth et al. conducted a systematic literature review of research that investigates the potentials of commercial exergames for rehabilitation and the improvement of physical health. Analyzing 20 empirical studies, they report promising evidence for positive effects of playing on quality of life and physical health. Although more research is still needed, it can be concluded that commercial exergames can usefully complement conventional rehabilitations measures.

Exergames are one example of how gaming technologies can be used to enhance people's lives and to teach useful skills. Another example is the many serious games that have been developed to help improving media literacy. Focusing on how media literacy is understood in such games and how such games are designed, Glas et al. conducted a thematic analysis of 100 games. The authors found that misinformation is the predominant theme, while other important topics, such as cyberbullying prevention or cybersecurity, are underrepresented.

However, players do not only learn from serious games designed to teach particular skills. Also, commercial entertainment games can serve as a source for informal learning. Vahlo et al. conducted a survey among 1,202 gamers from the UK and the USA and found that gaming can lead to a wide spectrum of learning outcomes. Further, it was found that learning outcomes were positively related to wellbeing as well as to eudaimonic gaming motivations (i.e., self-attributive motives like mastery or socializing that go beyond hedonic motivations, such as fun or relaxation).

Eudaimonia (in contrast to hedonia) also plays a central role in the theoretical piece by Possler et al.. They define meaningful or eudaimonic gaming experiences as “experiences that reflect human virtues and encourage players to develop their potential as human beings fully.” Their overview systematizes relevant theoretical approaches along four central game elements (social, narrative, agency, and aesthetics) and considers the degree of interactivity to explain how eudaimonic experiences develop in different types of games.

A good example of how gaming can become a meaningful, eudaimonic experience to players is provided by Eum and Doh. For their study, twelve individuals that had experienced the loss of a loved one were asked to play two different games that deal with the topic of death and remembrance. Drawing on game diaries and in-depth interviews, their study illustrates how playing these games can be a meaningful experience for the participants and help them to cope with their grief.

## The gray area in between

Of course, many aspects and effects related to gaming cannot be defined as simply light or dark. Rather, they have to be located somewhere in the large gray area between the two extremes.

For example, Kasdorf presents a qualitative analysis that deals with the representation of mental illness in video games published between 2018 and 2019. She argues that video games often portray psychological problems in a discriminatory and stigmatizing manner. However, some newer games by independent developers have taken a more balanced perspective on the topic that may help to counter stereotypes. Her findings and the category system she created could inspire further research on the topic and contribute to the development of games that offer a more empathetic and multidimensional portrayal of mental illness.

There has also been some progress in the representation of LGBTQ+ characters in video games in recent years: as discussed by Gaudszun and Elmezeny, the portrayal of queer protagonists in games has become more frequent, even if it is still too one-dimensional in many cases. Conducting an analysis of the social media communication surrounding two games that feature queer characters, the authors investigate the strategic communication of gaming companies and how they can successfully align with LGBTQ+ stakeholders. The results emphasize the importance of authenticity and a communication strategy that is in line with corporate practices, avoiding *rainbow washing*.

In general, it can be assumed that the way in which topics are presented and framed in video games can have an influence on our perception and thinking. Groen and Jacobs focused on persuasive games, i.e., games that were designed with the explicit intention of drawing our attention to specific real-world issues and shaping our attitudes. Their experimental study shows that games were more effective when the persuasive intent was clear. Further, playing intentions were stronger when the game was recommended by a peer (in contrast to system-based recommendations).

A specific form of persuasive games are advergames, i.e., games designed for marketing purposes. Cañete Sanz and De La Hera present a systematic review of research on advergames published between 2005 and 2021. They found that most academic studies in this area focus either on the effects of advergames on children (especially in terms of health and nutrition) or on purchase intentions, while studies on brand narratives and brand loyalty have been conducted less frequently.

Finally, based on statements of 180 players aged 15 to 25 years, Meriläinen and Ruotsalainen present an extensive analysis of the way young people use games, the meanings they attach to gaming and the effects they experience. Their results emphasize how complex young people's relationship to digital games can be and how many factors and aspects need to be taken into account when trying to understand them. It is difficult and probably misleading to frame gaming as an *either or* between beneficial and detrimental, but more holistic approaches are needed to better understand individual gaming experiences.

We hope that the articles published as part of this Research Topic will contribute to a more diverse picture of the many different facets of digital games and will stimulate a fruitful dialog among researchers.

## Author contributions

FR: Conceptualization, Project administration, Writing – original draft, Writing – review & editing. MS: Conceptualization, Writing – review & editing. TD: Conceptualization, Writing – review & editing.
